# Guided Stochastic Optimization for Motion Planning

**DOI:** 10.3389/frobt.2019.00105

**Published:** 2019-11-12

**Authors:** Bence Magyar, Nikolaos Tsiogkas, Bruno Brito, Mayank Patel, David Lane, Sen Wang

**Affiliations:** ^1^School of Engineering and Physical Sciences, Edinburgh Centre for Robotics, Heriot-Watt University, Edinburgh, United Kingdom; ^2^Department of Mechanical Engineering, KU Leuven, Heverlee, Belgium; ^3^Core Lab ROB, Flanders Make, Heverlee, Belgium; ^4^Department of Cognitive Robotics, Delft University of Technology, Delft, Netherlands; ^5^Fraunhofer IPA, Stuttgart, Germany

**Keywords:** motion planning, trajectory optimization, learning from demonstration, robot manipulation, robot learning

## Abstract

Learning from Demonstration (LfD) is a family of methods used to teach robots specific tasks. It is used to assist them with the increasing difficulty of performing manipulation tasks in a scalable manner. The state-of-the-art in collaborative robots allows for simple LfD approaches that can handle limited parameter changes of a task. These methods however typically approach the problem from a control perspective and therefore are tied to specific robot platforms. In contrast, this paper proposes a novel motion planning approach that combines the benefits of LfD approaches with generic motion planning that can provide robustness to the planning process as well as scaling task learning both in number of tasks and number of robot platforms. Specifically, it introduces Dynamical Movement Primitives (DMPs) based LfD as initial trajectories for the Stochastic Optimization for Motion Planning (STOMP) framework. This allows for successful task execution even when the task parameters and the environment change. Moreover, the proposed approach allows for skill transfer between robots. In this case a task is demonstrated to one robot via kinesthetic teaching and can be successfully executed by a different robot. The proposed approach, coined Guided Stochastic Optimization for Motion Planning (GSTOMP) is evaluated extensively using two different manipulator systems in simulation and in real conditions. Results show that GSTOMP improves task success compared to simple LfD approaches employed by the state-of-the-art collaborative robots. Moreover, it is shown that transferring skills is feasible and with good performance. Finally, the proposed approach is compared against a plethora of state-of-the-art motion planners. The results show that the motion planning performance is comparable or better than the state-of-the-art.

## 1. Introduction

Motion planning for manipulation tasks in human environments is a challenging problem. Even tasks deemed trivial, such as picking from shelves or opening drawers and cupboards, as described by Wurman and Romano (2015) and Jain et al. (2010), often require task-specific mathematical models or scripting. In fact, the most common pattern is using hand-crafted segments: a motion plan to a pre-grasp position and an approach movement, a number of segments to imitate a task, and a depart movement as demonstrated in competitions as recently reported by Correll et al. (2018). These systems are usually carefully crafted to the task and environment at hand, which does not allow for generalizing and scaling (Stilman, 2010).

Robots employed in warehouse and home environments are beginning to face increasing complexity in both task and environmental settings. Given that such environments were constructed to be occupied and used by humans, it can increase the difficulty for a robot to complete a given manipulation task. As a measure to overcome this problem, the state-of-the-art in collaborative robots often supports compliant behaviors and leverages *Learning from Demonstration* (LfD) approaches to allow adapting to new tasks or environments. In fact, LfD offers a solution for manipulation problems by using prior demonstrations without explicitly modeling the environments and systems. This alone, however, is not enough to handle changing parameters of a task. For example, such an approach does not allow picking from a different position on a shelf or opening a different set of drawers. Moreover, such LfD approaches are usually bound to the specific platform, meaning that using different robots or robot types to perform the same task would require to train each robot separately. Such an approach cannot scale to large heterogeneous teams of robots performing multiple tasks, where each of the task must be taught to each robot. For example, given the state-of-the-art and the variety of industrial robots offered by multiple manufacturers, independent teaching can be costly. This can reduce the flexibility of quickly allocating different tasks to different robots, when they are not trained for that particular task. In addition, such practices can lead to vendor lock-ins once a company starts using a specific task representation to train their collaborative robots.

**Figure 1:**
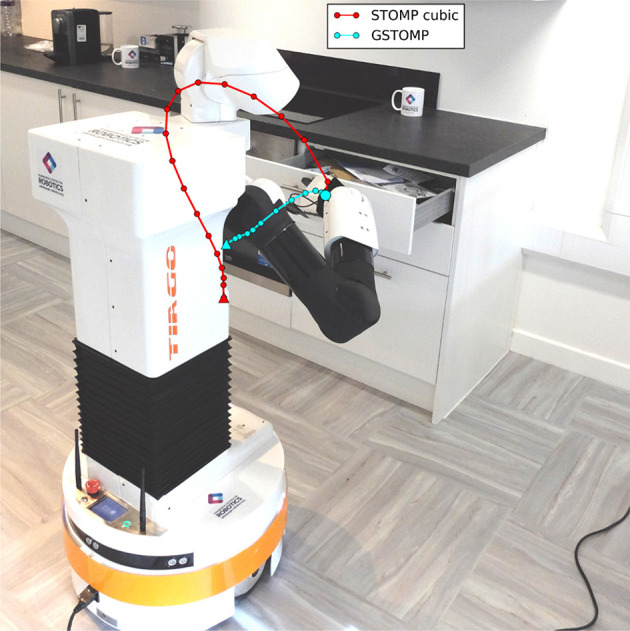
Planning for opening a drawer: STOMP cubic and GSTOMP trajectory.

Additionally, these LfD approaches are unable to guarantee collision-free planning. Once a trajectory is learned, they are able to follow it precisely but ignore the state of the environment. Such an approach requires the environment to be static to be successful. On the other hand, using a mobile manipulator, as the one seen in Figure 1, inherently involves uncertainty given errors in localization of the mobile base. Moreover, a household environment is usually changing from day to day and the robot has to operate near humans. For these reasons the success of each task requires a successful motion plan.

To address the aforementioned issues this work proposes the use of a *Dynamical Movement Primitive* (DMP) based approach. DMPs are a flexible way to encode the characteristics of a trajectory in a way that can be used to perform the same task in different conditions. In addition, DMPs can be implemented in Cartesian-space, making them platform-invariant. A trajectory trained in one robot can be easily reproduced by another robot making knowledge transfer easy. For solving the motion planning problem an improved variant of the *Stochastic Trajectory Optimization for Motion Planning* (STOMP) (Kalakrishnan et al., 2011) is used. In general, STOMP has been a successful and popular approach to solving the motion planning problem. Being as versatile as stochastic optimization, STOMP lacks the ability to handle tasks more complex than *A-to-B* planning and may also suffer from local minima depending on the initial guess. These factors may degrade performance of both speed and success of planning. It would be ideal to start from trajectories that already reach the goal from the start state such that STOMP only needs to make small adjustments (if any) to avoid collisions and joint limits.

The proposed novel algorithm, termed *Guided Stochastic Optimization for Motion Planning* (GSTOMP), introduces the use of task-informed trajectory initialization and a new cost function to the optimization. Task-informed trajectory initialization is essential to successfully perform the specific task, but can also help the motion planning process. The importance of trajectory initialization for STOMP and other optimization-based planners was observed and well noted in the work of Schulman et al. (2014). It is mentioned that such methods may require multiple initializations or task-informed initialization to improve their success rate. This work uses the latter by introducing *guide trajectories* encoded through DMP as *initial trajectories* for STOMP. Such a representation makes it easy to represent task specific constraints without an explicit mathematical formulation, which can be hard to model. A new cost function is used to penalize moving away from the *guide trajectory* (i.e., the one generated by DMP as explained in section 3.1) in Cartesian-space while still providing obstacle and joint limit avoidance. Experimental results show that introducing a LfD initialization method, increases the success rate of the planner and allows specific tasks to be successfully completed. The main contributions are the following:

1. A new initialization strategy is introduced which is able to bring task-specific constraints without explicit, task-specific mathematical formulation for STOMP.
2. A novel cost function is proposed extending the joint-space optimization of STOMP with Cartesian-space properties.
3. Skill transfer is performed between two robots, demonstrating the specific capability of GSTOMP.
4. An experimental validation of GSTOMP robustness to *Degrees of Freedom* (DoF) changes in the manipulator system compared to the state-of-the-art in motion planning tasks.
5. An extensive experimental validation of GSTOMP as a motion planner on two real manipulators and three different planning scenarios. It is shown that it achieves similar or better results than the state-of-the-art.

The proposed approach is evaluated in simulation and using real robots. Evaluation is performed using three different perspectives. The first one is based on task execution success: it aims to show the benefits of the proposed approach over state of the art methods. The second evaluation perspective is based on task execution success of multiple platforms. A task is taught using one platform and then others perform the same task. This metric aims at evaluating the success and benefits of skill transfer. The final evaluation perspective focuses on the proposed approach as a generic A-to-B motion planner. This aims to provide information regarding the competence of the proposed approach at finding generic motion plans. In all three perspectives the performance of the proposed approach is equal or better than the state of the art.

The rest of this paper is structured as follows. The relevant state of the art is presented in section 2. Section 3 introduces theoretical background for the components of the proposed approach. Evaluation and comparison with other methods are presented in section 4. Finally, section 5 concludes the paper offering potential paths for future study.

## 2. Related Work

RRTs (RRTsimples) by Kuffner and LaValle (2000) are state-of-the-art planning algorithms highly efficient and widely used for low-dimensional problems. RRTsimples excel at exploring large search spaces thus are often employed for path-planning on mobile robots, autonomous cars or robotic arms. Unless specifically instrumented however, RRTsimples are also known to produce jerky or overly elongated, suboptimal paths.

Experience-based planning with *sparse roadmap spanners* introduced by Coleman et al. (2015) relies on a large *experience database* of pre-recorded body trajectories with a given robot. It aims to use these as a start of the optimization and *repair* them according to a new scene. These experiences are used to encode solutions to some computationally expensive checks, such as joint limits, self-collision, and stability constraints. Similarly, GSTOMP uses DMPs to encode such information from a single demonstration. Roadmap spanners focus on high degree of freedom, highly constrained systems such as bipedal humanoid robots and works with experiences recorded in joint space while GSTOMP focuses more on arm motion planning by means of pre-recorded Cartesian-space trajectories and stochastic optimization.

Reachability Maps and Inverse Reachability Maps were explored by Zacharias et al. (2008) and Vahrenkamp et al. (2013), respectively. Both works explore the placement of a mobile manipulator to execute stored Cartesian trajectories for the task of drawer opening and pick and place, respectively. Despite targeting similar tasks as this work, the main focus on these approaches is on a single task at a time. They try to solve each task by placing the mobile base in an appropriate position that would allow the execution of a fixed task-specific trajectory. Opposite to what many other methods in the literature do, these two approaches do not focus on generating the trajectory required to execute the task.

*Covariant Hamiltonian Optimization for Motion Planning* (CHOMP) by Zucker et al. (2013) is an optimization-based approach which—similarly to STOMP—works by iteratively improving an initial trajectory along a cost function responsible for smoothness and obstacle avoidance. Building on CHOMP the work presented by Osa et al. (2017) uses CMA-ES and a distribution of demonstrations to learn cost functions for CHOMP. This work presents a similar approach to ours with appealing results in a task of disentangling a rope while avoiding collision with objects. However, it needs a rich set of joint-space demonstrations for every task.

The work of Marinho et al. (2016) proposes a joint-space trajectory representation using Reproducing Kernel Hilbert Spaces. They elaborate on the optimization process and update rules with respect to smoothness and obstacle avoidance constraints and show to perform better than CHOMP in a simulated scenario. There is a strong motivation for using such representations as they make reasoning about smoothness, described by acceleration, jerk, snap etc., trivial. Unfortunately, the many open parameters make this method hard to tune for any practical application. The authors propose using different kernels which all come with their respective parameters on top of the parameters of the proposed optimization method. Unfortunately, there was no suggested method to tune the parameters making it hard to apply in a different problem or even replicate the results. Moreover, adding additional constraints in a gradient-dependent system requires adapting the optimization process itself.

SBPL by Cohen et al. (2010) is a search-based method where a graph is built by using atomic motions referred to as motion primitives. The primitives in SBPL serve a guiding role, exploring the state-space reachable by the robot kinematics. However, they are only static building blocks, being limited to a certain set of moves. Compared to DMPs they lack flexibility and only encode single steps rather than entire trajectories.

Ijspeert et al. (2013) present a time-independent, scalable trajectory representation coined DMPs that allow start and end states to be changed while maintaining the dynamic characteristics of the motion used as demonstration. DMPs are able to represent motion in either joint-space or Cartesian-space although rotations in the latter case require special attention as discussed in Ude (1999) and Kramberger et al. (2016).

Policy Improvement with Path Integrals (*PI*2) in Theodorou et al. (2010) is a probabilistic learning algorithm with a single parameter as exploration noise. It scales well to high dimensions and ideal for optimizing joint-space DMPs. The work presented in Stulp et al. (2012) used *PI*2 for learning policies over sequences of DMPs for grasping under uncertainty to solve pick and place tasks. A thorough review of the family of algorithms including CMA-ES, *PI*, *PI*2, *PI*^*BB*^ and STOMP is presented in Stulp and Sigaud (2013).

For DeBaTo (Koert et al., 2016) employ *Probabilistic Motion Primitives* (ProMPs) in a Relative Entropy Policy Search framework for obstacle avoidance and trajectory optimization. Similarly to GSTOMP, a method to compare generated trajectories to a set of demonstration trajectories was used during the optimization process. This approach featured the Kullback–Leibler Divergence (KL divergence) as distance measure in the optimization against the set of demonstrations. However, some of the demonstration constraints do not fit within the scope as KL divergence is distribution-based.

The method proposed by Rana et al. (2017) integrates ProMPs within the CHOMP framework. Motivated by similar challenges as GSTOMP they adopt a probabilistic approach to motion representation in joint space however due to the adopted probabilistic approach, they require several different demonstrations of the task. While this approach performs well in the experiment they perform, unfortunately it is strongly tied to a joint-space distribution representation, therefore to the robot platform and setup the training was performed on. It is hard to see how the method could achieve skill-transfer over different platforms.

The method proposed in Kyrarini et al. (2017) uses GMMs (GMMs) to learn a motion from several human demonstrations. Similarly to the proposed approach they used kinesthetic teaching to record the demonstrations. Despite the presented results that method was demonstrated in a setup tailored to solving a specific task. Unfortunately, there was no variation to the task being solved to show that it can generalize well. In addition, that approach is a collection of preexisting tools instrumented to solve that specific task, adding small value to the state-of-the-art. In comparison, the proposed approach of this work requires only a single demonstration for learning a task. Moreover, it shows that it can generalize well solving multiple tasks of the same type and it can even transfer knowledge between different systems. Finally, the proposed approach presents a novel method of incorporating LfD techniques in a robust state-of-the-art motion planning method.

Although our work does not address the methods of capturing demonstrations and grasp planning, they form an important role in tackling the manipulation problem. Since the GSTOMP framework uses Cartesian-space motions to represent manipulation skills, many existing techniques can be leveraged to acquire new skills. Kinesthetic teaching is used in our experiments to acquire demonstrations, but the same technique can be used to learn from other planners, motion capture systems or video analysis. A unified representation for motion is proposed in Mandery et al. (2016): the output of marker-based motion-capture is converted into a unified representation called the *Master Motor Map* which converts the motion to any humanoid robot known to the system. We believe that grasp planning is core to the problem of motion planning for manipulation and its importance needs to be emphasized. As demonstrated in Yang et al. (2015) a popular state-of-the-art approach is able to learn grasp types corresponding to objects from readily available video material by using Convolutional Neural Networks.

In light of previous work, we decided to use a combination of described methods to balance benefits and limitations for our use case. In the following section, we introduce the theoretical framework of the GSTOMP approach.

## 3. GSTOMP

**Figure 2:**
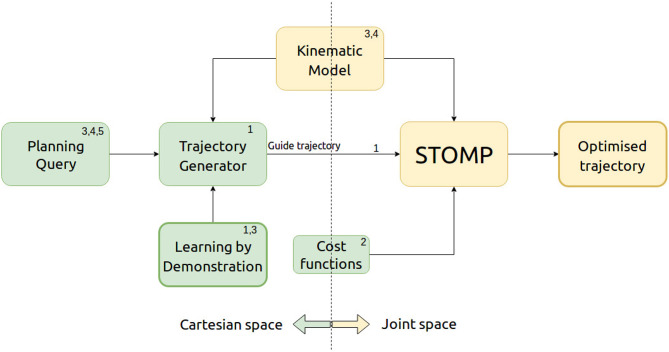
The architecture of GSTOMP. The numbers refer to our contributions presented in section 1. When the user requests a task to be performed the trajectory generator uses the kinematic model of the robot plus motion information learned by a demonstration. This trajectory is then used as an initial guide trajectory for STOMP to perform optimization using the robot's kinematic model and user defined cost functions. The outcome of this process is an optimized trajectory that can be used to successfully perform the requested task.

**Figure 3:**
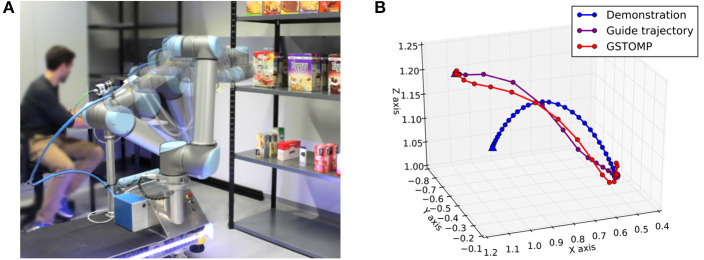
**(A)** Shelf picking experiment executed on the real *Rob@work* robot drawn with the *onion skinning effect*. **(B)** An example application of the GSTOMP algorithm. Initially the blue trajectory is demonstrated by a human for completing a specific task. The starting pose of the trajectory is denoted by the circle on the right side of the plot, while the ending pose of the trajectory is shown as a triangle on the left side. Then a new task is requested to be completed a guide trajectory is generated as shown by the purple line. This is finally optimized to the resulted red line. The hook-shaped artifacts are results of the different kinematic and joint-space smoothness constraints and are unique to this example.

GSTOMP combines the optimization framework of STOMP with a flexible trajectory generator implemented with Cartesian DMPs and a trajectory distance measure based on *Dynamic Time Warping* (DTW). Figure 2 depicts our architecture and draws references to our contributions list. The three steps of acquiring a solution within GSTOMP is shown in Figure 3B through an example. A demonstration was recorded reaching for a target. The new target is on the same shelf but located to the right from the previous one. Note how the shape is preserved in the *guide trajectory* and how the optimization modifies it, creating a robot trajectory. Circle and triangle markers mark the start and end states, respectively.

Note that since the DMP-generated *guide trajectory* is in Cartesian space and STOMP operates in joint space, a one-time conversion is necessary. TRAC-IK by Beeson and Ames (2015) is employed in GSTOMP as it has been shown to outperform the popular KDL solver. For redundant chains both default to a pseudo inverse Jacobian but GSTOMP aims to keep it robot-independent, hence the use of a general IK solver library. The rest of this section briefly introduces each component.

### 3.1. Flexible Trajectory Generator

A popular approach for generating trajectories from demonstrations in a flexible way is *Dynamical Movement Primitive* (Ijspeert et al., 2013). Specifically for our case Cartesian Dynamical Movement Primitives (DMPs) (Kramberger et al., 2016) are used, which can be defined as combination of position and rotation components (DMP_pos_, DMP_rot_). The necessity of separating the position component from the rotational part lies in the fact that rotations cannot be reliably represented by ℝ^3^ but have to be either in unit quaternion or rotation matrix form as explained in Ude (1999).

The position DMP component is described by the damped spring model as shown in the following equation:

(1) $$\begin{array}{l} \tau \ddot{y}=\alpha_{z}\left(\beta_{z}\left(g-y\right)-\dot{y}\right)+f \end{array}$$

where *y* is the current position, τ is a scaling factor for time, *g* is the goal position. α_*z*_ and β_*z*_ are scaling terms and *f* is a forcing term. The forcing term *f* is defined as:

(2) $$\begin{array}{l} f\left(x\right)=\frac{\sum_{i=1}^{B}\Psi_{i}\left(x\right){\text{w}}_{i}}{\sum_{i=1}^{B}\Psi_{i}\left(x\right)}x\left(g-y_{0}\right) \end{array}$$

where *y*_0_ stands for the initial state while *g* is the goal state, *B* is the number of basis functions, **w**_*i*_ is weighting for a given basis function **Ψ**_*i*_. While *B* is user defined, **w** is learned from demonstration. The time element in this formula is represented by its own first order linear dynamics such that

(3) $$\begin{array}{l} \tau \dot{x}=-\alpha_{x}x \end{array}$$

where α_*x*_ is a constant. **Ψ**_*i*_(*x*) is exponential basis functions:

(4) $$\begin{array}{l} \Psi_{i}\left(x\right)=\exp\left(-\frac{{\left(x-{\text{c}}_{i}\right)}^{2}}{2\sigma_{i}^{2}}\right) \end{array}$$

where σ_*i*_ and **c**_*i*_ define the width and centers of the basis functions, respectively. For a thorough discussion of the characteristics, implementation and training of DMPs we kindly refer the reader to Ijspeert et al. (2013).

Regarding the rotation DMP component, it is known that no minimal representation of orientation exists in *R*^3^ that contains no singularities and that its differentiation is numerically stable.

To solve this problem, Ude (1999) proposes solutions either by using rotation matrices or unit quaternions. GSTOMP employs quaternion DMPs in its Cartesian DMP implementation.

However, a quaternion is a non-minimal representation as **q** ∈ ℝ^4^. Consequently, this DMP formulation cannot be directly used as it assumes independent numerical values for each degree of freedom.

According to Kramberger et al. (2016) (Equation 1), can be rewritten in quaternion format as two equations, one to cover the original acceleration-space damped spring equation, and one in velocity space as

(5) $$\tau \dot{\eta }=\alpha_{z}\left(\beta_{z}2\text{log}\left(g_{o}*\bar{q}\right)-\eta +f_{o}(x)\right)$$

(6) $$\begin{array}{l} \tau \overset{∙}{\text{q}}=\frac{1}{2}\eta *\text{q} \end{array}$$

where η ∈ ℝ^3^ is the angular velocity vector, * denotes the quaternion multiplication and *f*_*o*_ is the equivalent of *f* for quaternions. Note how the goal term changed to a quaternion difference which is defined by multiplication by the conjugate. The logarithm of this difference returns an angular velocity vector, where the logarithm of a quaternion such that log:𝕊^3^ → ℝ^3^ is defined below:

(7) $$\text{log}(q)=\text{log}(v+u)=\{\begin{array}{ll} \text{arccos}(v)\frac{u}{\left\|u\right\|}, & \left\|u\right\|\neq 0\\ 0\in {\mathbb{R}}^{3}, & \text{otherwise} \end{array}$$

where **q** can be deconstructed into a scalar part (*v*) and a vector part (**u**).

The DMP function approximator requires accelerations therefore we differentiate the desired trajectory **q**^*des*^.

(8) $$\begin{array}{l} \eta_{t}^{\text{des}}=\frac{2\times \log\left({\text{q}}_{t}^{\text{des}}*{\overset{-}{\text{q}}}_{t-\Delta t}^{\text{des}}\right)}{\Delta t} \end{array}$$

Subsequently, velocities are differentiated to acquire accelerations as follows:

(9) $$\begin{array}{l} {\overset{∙}{\eta }}_{t}^{\text{des}}=\frac{\eta_{t}^{\text{des}}-\eta_{t-\Delta t}^{\text{des}}}{\Delta t}. \end{array}$$

The first elements are initialized to **0** as in the beginning the robot is not moving: $\eta_{des}\left(0\right)=0\in {\mathbb{R}}^{3}$, ${\overset{∙}{\eta }}_{des}\left(0\right)=0\in {\mathbb{R}}^{3}$. Finally, the target values to fit a function to can be summarized as

(10) $${\text{f}}_{\text{target}}=\dot{\eta }-\alpha_{z}(\beta_{z}2\text{ log}(q_{o}*{\bar{q}}_{j})-\eta_{j}).$$

To generate a new trajectory Equation (5) is integrated. For integrating Equation (6), the inverse of the *log* operator is applied. The exponential of an angular velocity vector is such that *exp*:ℝ^3^→𝕊^3^ defined below:

(11) $$exp(r)=\{\begin{array}{ll} \text{cos}\left(\left\|r\right\|\right)+\text{sin}\left(\left\|r\right\|\right)\frac{r}{\left\|r\right\|}, & \left\|r\right\|\neq 0\\ 0\in {𝕊}^{3}, & \text{otherwise} \end{array}$$

### 3.2. STOMP Foundations

STOMP uses an initial guess for the optimization process. This has to be a joint-space trajectory driving the arm from start state to goal state. Three different initialization strategies are available currently: linear interpolation, cubic interpolation and a minimal control cost trajectory. In our algorithm description this initial trajectory is denoted as θ_guide_. Part of our main contribution is to allow trajectories generated by Cartesian DMPs to be used as θ_guide_ after being converted to joint-space.

Algorithm 1 presents the proposed GSTOMP algorithm, as an extension of STOMP. As presented in Kalakrishnan et al. (2011), *N* is the number of waypoints representing the trajectory, *A* is a finite differencing matrix which produces accelerations $\ddot{\theta }$ when multiplied by the state vector θ. *M* is a smoothing matrix used in the update step of the optimization, N is a set of normal distributions to sample noise from, while *S* and *P* denote the per-timestep cost and probabilities of each noisy trajectory, respectively. ${\bar{\theta }}_{k,i}$ denotes the *i*-th state in ${\bar{\theta }}_{k}$ and θ_*i*_ denotes the *i*-th state in θ, dof stands for the number of *Degrees of Freedom* (DoF) of the kinematic chain.

**Algorithm 1:** The GSTOMP algorithm

Given: - Start (*x*_0_) and goal (*x*_*N*_) states, $x_{i}\in {\mathbb{R}}^{\text{dof}}$ - A discretized trajectory $\theta_{\text{guide}}\in {\mathbb{R}}^{\text{dof}\times N}$ - A state-dependent cost function *q*(*x*_*i*_) Precompute: - *A* = finite difference matrix - *R*^−1^ = (^*A*^*T*^*A*) − 1^ - *M* = *R*^−1^, with each column scaled such that the maximum element is (1/*N*) - Let θ = θ_guide_ Repeat until convergence of trajectory cost *Q*(θ, θ_guide_): 1) Create *K* noisy trajectories, ${\bar{\theta }}_{1}\ldots .{\bar{\theta }}_{K}$ with parameters θ + *e*_*k*_ where $e_{k}=N\left(0,R^{-1}\right)$ 2) For *k* = 1…*K, i* = 1…(*N* − 1) compute: a) $S\left({\bar{\theta }}_{k,i}\right)$ = $q\left({\bar{\theta }}_{k,i}\right)$ b) $P\left({\bar{\theta }}_{k,i}\right)$ = $\frac{e^{-\frac{1}{\lambda }}S\left({\bar{\theta }}_{k,i}\right)}{\overset{K}{\underset{l=1}{\sum }}\left[e^{-\frac{1}{\lambda }S\left({\bar{\theta }}_{l,i}\right)}\right]}$ 3) For *i* = 1…(*N* − 1), compute: ${\left[\delta \bar{\theta }\right]}_{i}$ = $\overset{K}{\underset{k=1}{\sum }}P\left(\theta_{k,i}\right){\left[e_{k}\right]}_{i}$ 4) Compute δθ = M$\bar{\delta \theta }$ 5) Update θ ← θ + δ θ 6) Compute *Q*(θ, θ_guide_) according to Equation (17)

### 3.3. Cartesian Trajectory Cost Function

Providing a *guide trajectory* that looks like a good solution is not a guarantee for the trajectory to be maintained during the optimization process. To motivate exploration close to the *guide trajectory* we introduce a cost function on the similarity between the *guide* and optimized trajectories. DTW Sakoe and Chiba (1978) originated from natural language processing is a robust distance measure for two sets of time-series data. In contrast to existing approaches (Osa et al., 2017), our DTW implementation follows the original equations with a distance function on the Cartesian trajectory. This distance is defined by a weighted sum of the Euclidean distance of the position components and the quaternion logarithm error (used in Equation 5) of the orientation components. The distance function used in the DTW algorithm (Sakoe and Chiba, 1978) is defined as

(12) $$\begin{array}{l} d\left(i,j\right)={\text{w}}_{pos}∥{\text{p}}_{i},{\text{p}}_{j}∥+{\text{w}}_{ori}2∥log\left({\text{o}}_{i}*{\overset{-}{\text{o}}}_{j}\right)∥ \end{array}$$

where *i, j* are indices, each traversing a different trajectory, e.g. **p**_*i*_ and **o**_*i*_ denote the position and orientation, respectively of the *i*-th element in the first trajectory, *j* works similarly on the second trajectory. We empirically found $\frac{{\text{w}}_{pos}}{{\text{w}}_{ori}}=4$ to be ideal. This compensates for the numerical differences of the two distance metrics. For brevity, we refer to our Cartesian-space DTW implementation as *DTW* ∈ θ × θ → ℝ and is defined as the sum of element-wise time-warped distances:

(13) $$\begin{array}{l} DTW\left(\theta_{A},\theta_{B}\right)=\Sigma_{i\in Ind\left(\theta_{A}\right),j\in Ind\left(\theta_{B}\right)}d\left(i,j\right) \end{array}$$

where *Ind*(θ) denotes the indices of trajectory θ.

### 3.4. GSTOMP Cost Function

The composed cost function of GSTOMP takes collision checks, collision proximity and similarity to initial trajectory θ_guide_ into account by calculating a weighted sum of these components.

A collision checker function which assigns high values to states with collisions defined as follows,

(14) $$\xi (\theta_{i})=\{\begin{array}{ll} \text{collision}\_\text{penalty}, & \text{collisionWithWorld}(\theta_{i})\\ \; & \text{or collisionWithSelf}(\theta_{i})\\ 0, & \text{otherwise} \end{array}$$

where the boolean collision values are computed by collisionWithWorld(θ_*i*_) and collisionWithSelf(θ_*i*_) on a single node θ_*i*_ of the trajectory for collisions with the environment and the robot body, respectively. When used with an entire trajectory θ as a parameter, ξ ∈ θ → ℝ^*N*^, the function returns a vector of collision flags.

The following function defines a single collision gradient computation.

(15) $$\upsilon (\theta_{i})=\{\begin{array}{ll} 0, & \text{collisionDistance}(\theta_{i})\\ \; & >{\text{min}}_{\text{distance}}\\ \frac{{\text{min}}_{\text{distance}}-\text{collisionDistance}(\theta_{i})}{{\text{min}}_{\text{distance}}}, & \text{0 < collisionDistance}(\theta_{i})\\ \; & >{\text{min}}_{\text{distance}}\\ \text{collision}\_\text{penalty}, & \text{collisionDistance}(\theta_{i})<0 \end{array}$$

with collisionDistance(θ_*i*_) denoting the computation of the distance of any robot body part to the environment and itself, this is provided by the Flexible Collision Library presented by Pan et al. (2012). In our experiments, the value of min_distance_ was set to 0.2 m. The function which explores neighboring states and assigns a cost based on the proximity of states with collisions can now be defined as

(16) $$\begin{array}{l} \Upsilon \left(\theta \right)=\Sigma_{i=1}^{N-1}\left(\upsilon \left(\theta_{i}\right)+\Sigma_{j=1}^{L}\upsilon \left(f\left(\theta_{i},\theta_{i+1},\frac{j}{L}\right)\right)\right) \end{array}$$

where *f* ∈ θ × θ × ℝ → θ is a linear interpolation method with a scalar parameter *s* ∈ [0, 1] determining the phase of transition between the first and the second trajectory argument, *N* is the number of nodes in the trajectory and *L* is the number of intermediate points to check which was set to 5 in our experiments.

Finally, the GSTOMP cost function is defined as *Q*(θ, θ_guide_), reflecting that this is a STOMP cost function employed in Algorithm 1 using both the working trajectory (θ) and the initial trajectory (θ_guide_). In our experiments we prioritize on collision avoidance first, *guide* following second, and staying far from colliding states last.

(17) $$\begin{array}{l} Q\left(\theta ,\theta_{\text{guide}}\right)=w_{c}\xi \left(\theta \right)+w_{cg}\Upsilon \left(\theta \right)+w_{dtw}DTW\left(\theta ,\theta_{\text{guide}}\right)+\frac{1}{2}\theta^{T}R\theta \end{array}$$

where ξ is the collision checker function from Equation (14) and Υ is a collision gradient function defined in Equation (16) and *DTW* is the Cartesian trajectory distance defined in section 3.3. The corresponding weighting factors are denoted by *w*_*c*_, *w*_*cg*_, and *w*_*dtw*_, respectively. The weights can then be tuned to work well with other cost function(s) within a STOMP setup which for our experiments was not necessary.

Supporting a large variety of tasks with a method that may require tuning parameters between tasks is a near impossible challenge. In our experience, STOMP and GSTOMP only require tuning a single time per collection of cost functions. Once the weighting is adjusted such that it balances all components to a similar order of magnitude, taking preferences into account, the weights do not need further adjustment.

## 4. Experiments

After introducing the theoretical framework, the proposed method is extensively evaluated using real and simulated robots. The evaluation is performed on three different aspects. For the first evaluation aspect the proposed approach is compared on task success against a simple LfD approach. This aims to show the benefits of the proposed approach over the state-of-the-art techniques used in collaborative robots. The second aspect focuses on skill transfer among various robots. It shows how well the method performs when trained on one robot and then executing on other different platforms. Finally, the proposed approach is compared against other planners from the literature. This aims at showing the value of the proposed approach as a generic motion planner.

In all three evaluation aspects three metrics are used from the set presented by Cohen et al. (2012) and presented in Table 1. The first metric is the success rate of finding a plan. This metric is the most important of any motion planner as it validates its correctness and usefulness. The second metric is the planning time. Such a metric is important as the robot has to operate in the real world and finding good plans in an online manner is essential. The final metric is the smoothness of the found plan. This metric is important as smooth motion puts less stress to the mechanical structure of the robot as well as it makes it easier for people in the environment to predict the robot's movement. The smoothness value is a cumulative function of the end effector's linear and angular accelerations. The accelerations are approximated using Second Order Central Difference Approximation. Section 4.1 presents the experimental setup. In section 4.2 the results of the proposed approach against a LfD approach are presented. Section 4.3 presents the results regarding the skill transfer capabilities of the proposed approach. Finally, section 4.4 details the results of the proposed approach compared in generic A-to-B planning against state of the art methods.

**Table 1:** Metrics used to evaluate each experiment.

**Metric**	**Description**
T	Planning time in s
*s*_*r*_	Success rate in %
$\ddot{\Theta }$	Smoothness in *m*/*s*^2^

### 4.1. Experiments Setup

We decided to focus on common manipulation tasks around a home or a warehouse. The tasks we chose to study are:

- Picking an item from a shelf (Wurman and Romano, 2015).
- Opening a drawer (Jain et al., 2010).
- Opening a cupboard (Jain et al., 2010).

The shelf-picking experiment uses the 6 DoF mobile manipulator robot from *Fraunhofer IPA* called *Rob@work* which was designed to perform light industrial tasks and features a vacuum gripper. The drawer and cupboard opening experiments were conducted using the 8 DoF mobile manipulator robot, *TIAGo* from *PAL Robotics* which was designed for Ambient Assisted Living and uses a parallel gripper. In addition to the aforementioned scenarios, GSTOMP is evaluated in two more cases. In the first case a trajectory learned by one robot is used to perform tasks using another robot showing skill transfer between platforms. In the second case the DoFs of the robot is reduced, showing the benefit of the *guide* trajectory making GSTOMP more robust on different platforms.

Each environment used in our experiments was set up with the 3D models of the corresponding robot and furniture available in the respective labs. Planning queries were made against relevant planners using a set of pre-defined start and end points assuming the same static position of each robot with respect to the furniture.

Demonstrations were acquired by kinesthetic teaching on the real robots using the real furniture. For instance, having trained on opening the top drawer with a real *TIAGo* robot, GSTOMP will use the same demonstration to generate *guide trajectories* for the other start-end positions for opening this drawer or other drawers.

When using a vacuum gripper, we can assume perfect grasping at the target positions. Using the parallel gripper one has some freedom in rotation around the handles as well as along the length of the handlebar. An effect of this was discovered during the kinesthetic teaching phase: the shape of cupboard opening demonstrations does not follow the curve traveled by the door handle. This is explained by experiencing a higher rate of end effector rotational change in the demonstration trajectory and some travel along the length of the handle as mentioned earlier.

All STOMP variants were set to use 20 samples from a trajectory throughout our experiments. It is possible to fine-tune the standard deviation of the distributions N that are used for generating noise on each joint in STOMP, as shown in Algorithm 1. The default values of these parameters are relatively large, using a standard deviation of 1.0. Using this setting we often experienced vastly different trajectories from *guides*, resulting in slow convergence and high failure rate. It is preferred to take much finer steps when planning in tight, collision-rich spaces. Consequently the default 1.0 standard deviation was replaced with 0.1 for each joint in our experiments, for all STOMP variants and GSTOMP alike. This ensures a better fitting exploration in the noisy trajectory rollout phase of STOMP and significantly increases the convergence rate of the optimization process.

All experiments were conducted using the *MoveIt!* framework (Sucan and Chitta, 2013) on a system with an Intel i7-4710MQ, 2.5GHz CPU, 8GB of RAM and running on Ubuntu 16.04. STOMP and our extensions were implemented in C++, while the DMP framework was implemented in Python. For validation on the two real robots the *joint_trajectory_controller* (*ros_control*; Chitta et al., 2017) was used to execute the generated plans.

### 4.2. Working With Task-Specific Trajectories

**Figure 4:**
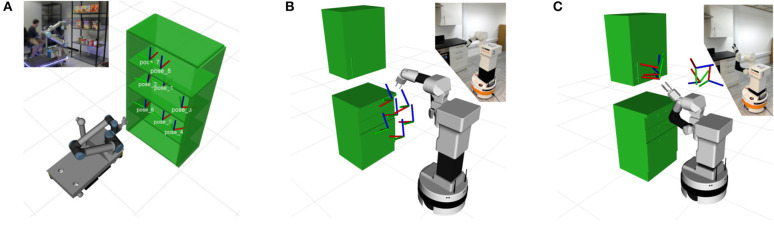
The scenarios used for verifying our approach. In the first scenario **(A)** the robot has to execute a picking and placing task in various positions of a shelf. The second scenario **(B)** requires various drawers to be opened. In the final, third **(C)**, scenario the robot has to open a cupboard. All the scenarios were tested in simulation and in real experiments with robots.

The first evaluation point is the successful task execution for three different tasks. The first task is a simple pick and place task as the ones used in the work of Wurman and Romano (2015). The second task involves opening a drawer while the third one focuses on opening a cupboard. Similar scenarios are used in the work of Jain et al. (2010). Examples of such tasks can be seen in Figure 4. The proposed approach is compared against a DMP-generated Cartesian *trajectory*, a simple LfD approach that represents the state-of-the-art in collaborative robots. Plans are validated for joint limits and collisions in the same framework without performing further optimization on it. Although it may be referred to as a planner in this experiments section, it serves as baseline for comparison.

**Figure 5:**
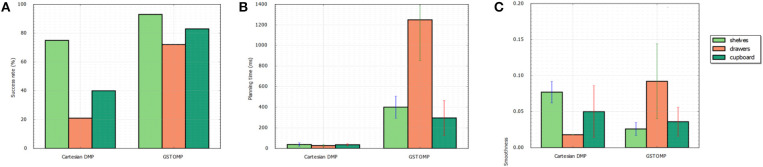
Comparison of the proposed approach with a learning from demonstration approach. Success rate **(A)** shows that the proposed approach performs better in any of the given tasks. This comes at the cost of planning time as it can be seen in **(B)**. Both methods perform in a comparable manner regarding the smoothness of the generated path **(C)**.

The results can be seen in Figure 5. For the first metric of the success rate the proposed approach is much higher than the LfD. In the pick and place case both methods perform well with the proposed approach being able to find a valid path in almost all the cases. The LfD approach is following with almost 80%. In the harder cases of opening a drawer or a cupboard the LfD approach performs much worse than the proposed approach. This shows the benefits of the proposed method over the state of the art approaches in collaborative robots.

Regarding the planning time metric the LfD approach is performing far better than the proposed method. It is much faster to rollout a DMP trajectory than to plan a full path and it is reflected on the results. Finally, both methods perform in a comparable manner in the smoothness metric. The proposed approach performs better in the shelves and cupboard scenarios, while the LfD approach performs better in the drawers scenario.

### 4.3. Skill Transfer

**Figure 6:**
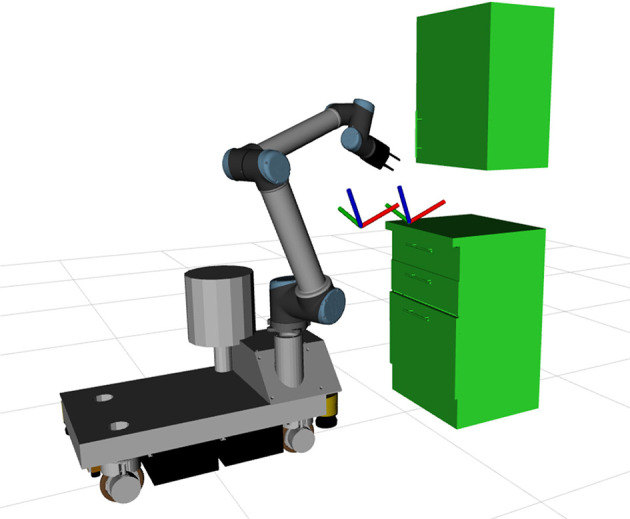
Experiment setup for transfer of drawer opening skill. The trajectory was trained using a *TIAGo* robot and is executed by the *Rob@work* platform.

**Figure 7:**
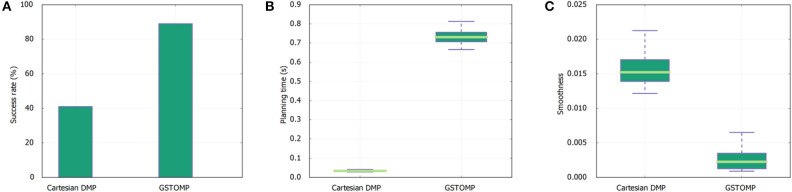
Results of skill transfer from the *TIAGo* robot to *Rob@work* platform. The success rate **(A)** is similar to the one presented in Figure 5 for the *TIAGo* robot. The LfD approach performs better in this platform but is still far from the good performance of GSTOMP. The planning time **(B)** favors the LfD while GSTOMP produces smoother plans **(C)**.

**Figure 8:**
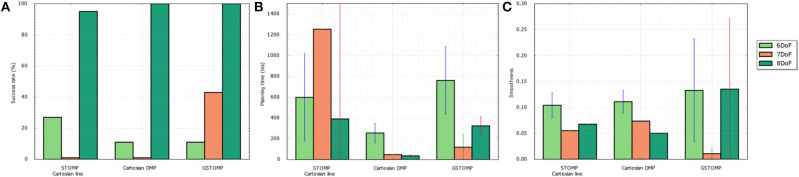
Success rate **(A)**, planning time **(B)**, and smoothness **(C)** for a single cupboard task with varying number of DoF enabled on the *TIAGo* robot. The proposed approach performs on par or better than most of the other approaches. It also shows a smooth degradation to the reduction of DoF.

The second evaluation point is the skill transfer capabilities of the proposed approach. For this a trajectory is learned using LfD with one robot and is tested using other robots. In the scope of this work a trajectory was learned using a *TIAGo* robot for the drawer opening task. It was then executed using the *Rob@work* platform, as well as, a *TIAGo* robot with reduced degrees of freedom. Limiting the degrees of freedom of a platform essentially turns it into a different platform. For this set of experiments the joints of the platform were set to a specific value and the optimization process was not allowed to change them. The experimental setup of the *Rob@work* platform can be seen in Figure 6, while results for both the experiments can be seen in Figures 7, 8.

Transferring skills between robots is a major merit of GSTOMP. The first set of results refer to the *Rob@work* platform executing tasks that were trained using a *TIAGo* robot. In this experiment we used the drawer opening skill demonstration recorded with *TIAGo* and executed it on the *Rob@work* platform. To provide even ground for comparison, an altered version was created of *Rob@work*, replacing the end effector with the parallel gripper of *TIAGo* in simulation. We chose drawer opening to demonstrate skill transfer due to the limited height of *Rob@work*.

For the first metric of the task success rate it can be seen that the proposed approach performs in a similar manner using the *Rob@work* platform and *TIAGo*. It is noteworthy that the success rate of the LfD approach is double for the *Rob@work* platform but still is more than half than the GSTOMP. In accordance to the previous results the planning time for the proposed approach is higher than the simple LfD approach in this platform as well. Finally, for smoothness the proposed approach performs better than the LfD approach for the *Rob@work* platform. This is in contrast to the results of the *TIAGo* platform.

For the second test some of the joints of *TIAGo* were set to a specific value and the optimization process was not allowed to change them. This aims to show the benefits of planning using GSTOMP with different manipulator systems having different numbers of DoF. In addition this experiment provides further reinforcement to skill-transfer between robots, as it is easy to consider that a robot with artificially reduced DoF is essentially a different platform.

In total 200 queries were made with the set of planners using three different DoF configurations of the same robot:

- The original, 8 DoF *TIAGo* robot consisting of the elevating torso and a 7 DoF arm,
- A fixed torso version, reducing the planning task to the 7 DoF arm, and
- A 6 DoF version of the arm, fixing the first joint such that it makes start and end states of the task possible.

The results for this tests can be seen in Figure 8. Regarding the task success it drops when the degrees of freedom are reduced as is expected. It can be seen that the proposed approach can perform 100% of the tasks when 8 DoFs exist. The same goes for the pure LfD approach, while STOMP with Cartesian initialization falls a bit behind. For 7 DoFs GSTOMP manages to perform 40% of the tasks while both of the other approaches are close to 0%. When the degrees of freedom are reduced even more STOMP performs better with 20% while the other two approaches are performing around 10% of the tasks. Regarding the planning time the LfD approach is consistently better. GSTOMP follows being statistically better than or equal to STOMP. Finally, regarding the smoothness metric, the proposed approach is better only for the case of 7 DoFs. In the other cases the other approaches perform better and in a more consistent given their variance.

### 4.4. General Planning

The last evaluation point of the proposed approach is its fit as a generic planner. For this it is compared against state of the art planners in finding paths using the aforementioned shelf, drawer and cupboard scenario. The planners used can be seen in the following list:

1. RRTConnect: a state of the art motion planner from *Open Motion Planning Library* (OMPL) (Sucan et al., 2012)
2. CHOMP: a planner similar to STOMP in nature
3. STOMP linear: STOMP using the linear joint-space interpolation initialization strategy
4. STOMP cubic: STOMP using the cubic spline joint-space interpolation initialization strategy
5. STOMP min control: STOMP using a minimal control joint-space interpolation initialization strategy
6. STOMP Cartesian line: STOMP using a linear interpolated straight Cartesian line as *guide* strategy
7. GSTOMP: the proposed approach of this work.

**Figure 9:**
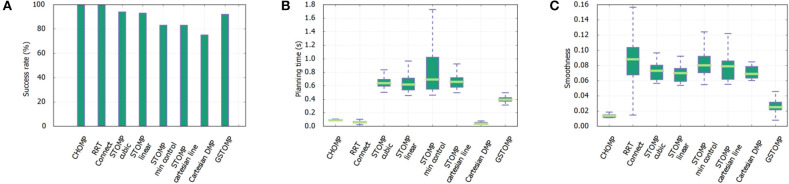
Shelf-picking scenario experiment results from 600 queries with each planner. The proposed approach performs close to perfect **(A)**, while requiring less time **(B)** than other STOMP variants. It also provides rather smooth paths only falling behind CHOMP **(C)**.

The results for the shelf-picking scenario are shown in Figure 9. This task provides an equal ground for all planners as is a simple *A-to-B* type of planning setup. Different target poses within shelves resemble randomly-placed objects on the three shelves. Our experiment consisted of 100 reaching and 100 retrieving queries for each of the three shelves. Retrieval queries were created by swapping start and end states of the reaching queries. A total of 600 queries were made against this scenario using one demonstration for each shelf as shown in Figure 4A. The proposed approach is on par with many of the state of the art planners only loosing to RRTConnect and CHOMP. Regarding planning time, again RRTConnect and CHOMP are preforming better, while all the other STOMP variants perform worse. Finally, the proposed approach is one of the smoothest with only CHOMP performing better. Figure 3 depicts the execution of a GSTOMP trajectory on the real robot.

**Figure 10:**
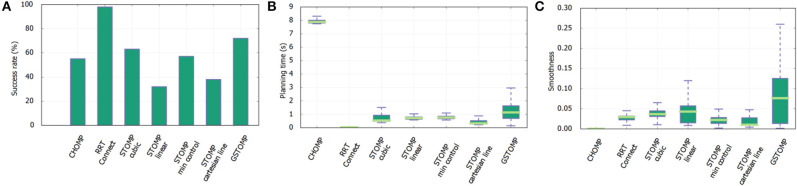
Drawer scenario experiment results from 600 queries with each planner. The proposed approach is performing better than most of the other approaches, coming second only after RRTConnect **(A)**. Regarding the planning time **(B)** the proposed approach is close to most of the other approaches, with exception of CHOMP which performs much worse. The proposed approach is statistically the worse performer in smoothness **(C)** but really close to all the other approaches.

The results for the drawer scenario can be seen in Figure 10. Regarding planning success RRTConnect performs the best, with the proposed approach coming second. The planning time requirements of the proposed approach are slightly higher than most of the other planners but still comparable. RRTConnect is again the best performer in this category. The bad performance of CHOMP is noteworthy in the planning time for this scenario. Finally, regarding smoothness the proposed approach performs statistically the worse compared to other approaches, with CHOMP performing the best. Sadly, despite CHOMP's outstanding performance on this metric, it's success rate is significantly lower than that of other planners. The performance of the proposed approach is in general close to the other planners.

**Figure 11:**
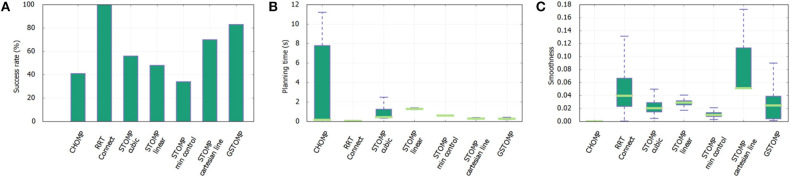
Cupboard scenario experiment results from 600 queries with each planner. The proposed approach is the second in success rate **(A)** only losing against RRTConnect. Regarding the planning time **(B)** the proposed approach is the second best after RRTConnect. Again CHOMP performs much worse. Finally, GSTOMP performs on par with other methods regarding smoothness **(C)**, with only CHOMP performing better.

Regarding the final scenario, shown in Figure 11, the proposed approach performs again second to the RRTConnect planner in terms of success. The planning time of most of the planners is comparable with RRTConnect and GSTOMP being the fastest, while CHOMP performing statistically the worst. In terms of smoothness CHOMP gives the best results with all the other planners performing close to each other. The worst performer was the STOMP initialized using a Cartesian line.

It should be noted how GSTOMP always performed as good as or better than other STOMP variants, clearly showing the benefit of the task-informed *guide* trajectory.

## 5. Conclusions and Future Work

In this paper a novel motion planning algorithm GSTOMP is proposed, which extends an existing optimization-based technique STOMP, to use trajectories (i.e., *guides*) for optimization initialization. These *guide* trajectories are produced by Cartesian DMPs. A new cost function is introduced to the optimizer that penalizes moving away from the *guide* trajectory unless it is overruled by other cost functions such as collision and joint limit avoidance. These appear as weighted terms in the optimization process of GSTOMP.

The system is able to generalize over single demonstrations of tasks for new start and goal states. The *guide trajectories* implicitly encode complexity without the need to design task-specific mathematical models or execution frameworks as in Stilman (2010).

The demonstrations used to generate *guide trajectories* may be acquired from kinesthetic teaching, motion capture systems or video analysis.

It is clear that approaches crafted for specific tasks are likely to perform better, but we are confident that GSTOMP scales well with the number of tasks. Extensive studies on shelf-picking and drawer- and cupboard opening scenarios were performed. The proposed method is compared against a variety of state-of-the-art approaches using the same software framework. GSTOMP is able to leverage prior demonstrations of tasks, ensuring correct, task-specific trajectories. This combination improved the success rate over pure LfD approaches. It allows the task specific trajectories to avoid obstacles and cope with variances in the environment.

It is also shown that GSTOMP allows for sharing *guide trajectories* between different robots achieving a form of skill-transfer. Since the demonstrations are captured in Cartesian-space, the only challenge for achieving this is kinematic feasibility: robots or humans may produce demonstration trajectories impossible to be tracked by a robot with fewer degrees of freedom or a different kinematic setup. A study on the effects of limited DoF is conducted which confirmed that GSTOMP is performing in such cases.

The parameters for joint-space noise distributions used in the *noisy trajectory generation* step of STOMP are currently determined empirically. When learning a task for a given robot, a set of trajectories could be used to learn these parameters.

Our method does not cover approach and departure paths as this can be achieved by any planner but rather focuses on generating task-specific trajectories.

The current approach uses sampling-based continuous collision checking. In the work of Schulman et al. (2014), the concept of *swept out volumes* is introduced. An interesting future direction would be to compare the two collision checking methods.

The source code of this project is planned to be released as a part of the official release of the STOMP software package.

Finally, in the current implementation the robot mobile base is assumed to be static in front of the task area. Inspiration could be taken from Vahrenkamp et al. (2013) and Zacharias et al. (2008) to add mobile base placement to the motion planning and initialization problem, further improving its success rate, computation time and smoothness.

## Data Availability Statement

All datasets generated for this study are included in the manuscript/supplementary files.

## Author Contributions

BM, BB, and NT contributed conception and design of the work. BM, BB, and MP implemented algorithms and benchmarking. BM and NT performed experiment analysis. BM, BB, and MP wrote the first draft of the manuscript. BM, NT, DL, and SW wrote sections of the manuscript and contributed to several revisions and restructuring. All authors contributed to manuscript revision, read, and approved the submitted version.

### Conflict of Interest

The authors declare that the research was conducted in the absence of any commercial or financial relationships that could be construed as a potential conflict of interest.
